# Adequacy of Clinical and Radiological Evidence for the Management of Kartagener Syndrome

**DOI:** 10.7759/cureus.91091

**Published:** 2025-08-27

**Authors:** Himangshu Hazarika, Garima Talukdar, Anjan J Talukdar

**Affiliations:** 1 Internal Medicine, Primary Health Centre Bihmari Bongaon, Biswanath Chariali, IND; 2 Medicine, Primary Health Centre Champabati, Chirang, IND; 3 Internal Medicine, Gauhati Medical College and Hospital, Guwahati, IND

**Keywords:** clinico-radiologic criteria, diagnostic difficulties, kartagener syndrome (ks), primary ciliary dyskinesia (pcd), resource-limited setting, situs inversus totalis

## Abstract

Kartagener syndrome, a clinical variant of primary ciliary dyskinesia (PCD), is a rare autosomal recessive genetic disorder caused by defective motility of cilia lining the respiratory tract, fallopian tubes, and middle ear. This results in impaired mucociliary clearance and leads to recurrent upper and lower respiratory tract infections, chronic sinusitis, otitis media, and bronchiectasis.

We report a case of a 36-year-old woman with longstanding recurrent sinopulmonary infections, productive cough, hemoptysis, and dyspnea, who was ultimately diagnosed with left lower lobe bronchiectasis and situs inversus totalis. Bronchoscopy with bronchoalveolar lavage grew *Pseudomonas aeruginosa*. In the absence of advanced confirmatory tests for PCD, such as nasal nitric oxide measurement, saccharin test, high-speed video microscopy, transmission electron microscopy, and genetic analysis, the diagnosis was made on the basis of clinical and radiological findings alone.

This case underscores the practical challenges of diagnosing Kartagener syndrome in resource-limited settings with constrained access to advanced diagnostic tools, gold-standard genetic tests, specialized personnel, and comprehensive healthcare infrastructure. In our setting, access was limited to basic hematological and biochemical tests and a conventional chest X-ray. It further examines whether a strong clinico-radiological correlation can be sufficient to guide appropriate treatment and management in such contexts.

## Introduction

Kartagener’s syndrome, a subtype of primary ciliary dyskinesia (PCD), is a rare autosomal recessive disorder caused by defective motile cilia. These hair-like organelles line the respiratory tract, paranasal sinuses, middle ear, eustachian tubes, and fallopian tubes, playing a crucial role in mucociliary clearance and proper organ laterality during embryogenesis [1]. Impaired ciliary motility leads to ineffective clearance of airway secretions, predisposing patients to recurrent respiratory infections such as rhinosinusitis, otitis media, pneumonia, and bronchiectasis, often associated with pathogens like *Pseudomonas aeruginosa* [2]. Infertility due to impaired gamete transport is also common [1]. Approximately 50% of patients exhibit situs inversus, which forms the classic diagnostic triad of Kartagener’s syndrome: chronic sinusitis, bronchiectasis, and situs inversus [2].

Although diagnostic confirmation typically requires specialized investigations such as high-resolution imaging or electron microscopy, Kartagener’s syndrome can often be clinically suspected with careful history-taking, thorough physical examination, and judicious use of basic investigations, including chest radiography and electrocardiography [3]. In resource-limited settings, this clinical approach is invaluable, as advanced diagnostic tools may be unavailable. The present case highlights how trained clinical reasoning, supported by conventional imaging, enabled the suspicion of Kartagener’s syndrome, with CT and bronchoscopy serving a supplementary role to consolidate the diagnosis.

## Case presentation

A 36-year-old female presented to a government secondary health center with complaints of a productive cough for three weeks, associated with fever, blood in sputum, and intermittent exertional breathlessness. Her history revealed that she had experienced similar episodes of productive cough about two to three times annually since the age of 13, for which she had recurrent hospital visits and chronic use of antibiotics, but with no hospital admissions. She reported intermittent nasal obstruction and occasional purulent nasal discharge since adolescence, consistent with chronic rhinosinusitis. There was no history of recurrent or serous otitis media, ear pain, discharge, or hearing loss. There was no confirmed history of infertility, as the patient had been married for only 10 months and had been actively trying to conceive since marriage. However, given that infertility is clinically defined only after 12 months of unprotected intercourse without conception, it remained inconclusive at the time of evaluation.

**Figure 1:**
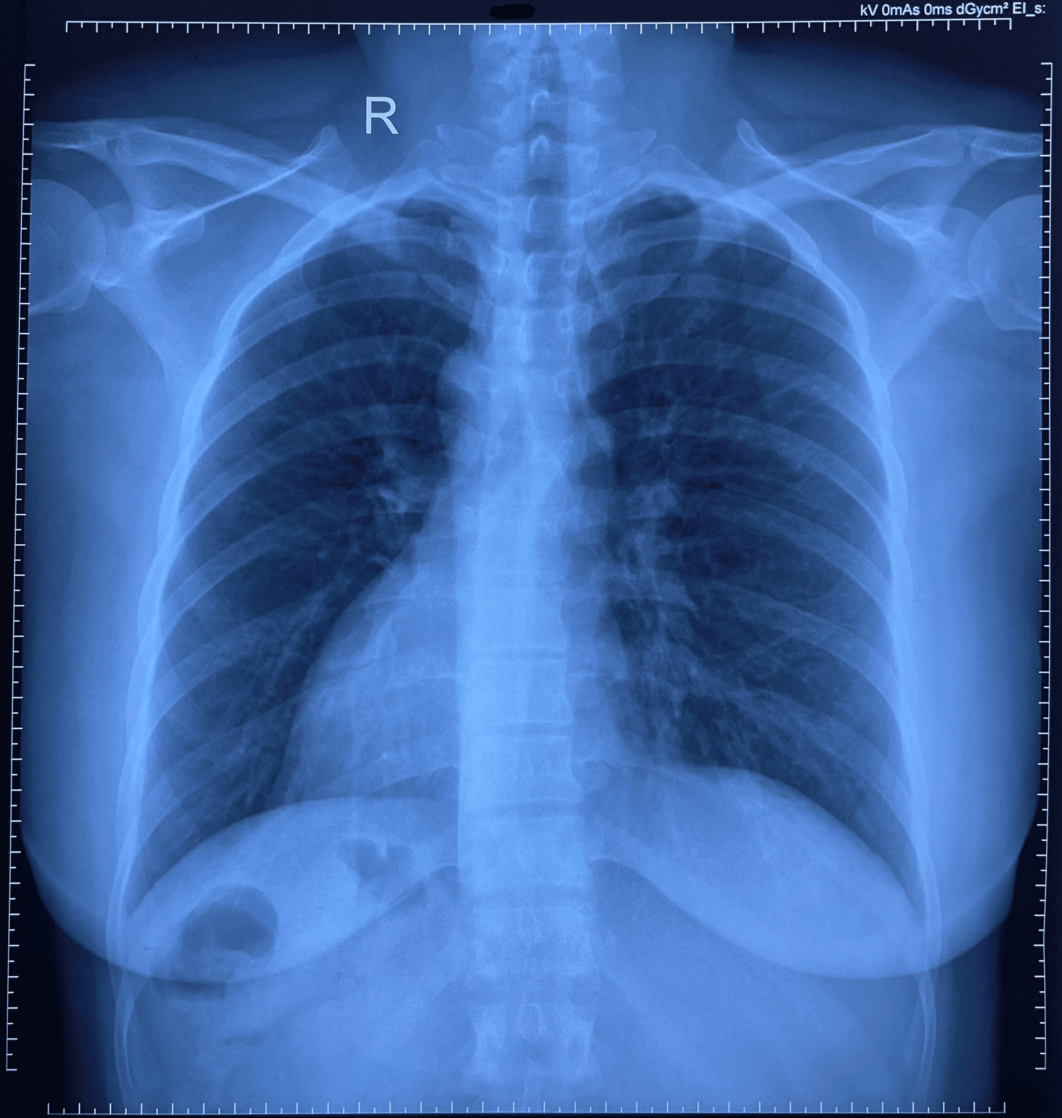
Chest X-ray (posteroanterior view). Chest X-ray (posteroanterior view) showing dextrocardia and reversal of abdominal organ positions, suggestive of situs inversus. The heart shadow is seen on the right side, and the gastric bubble is visualized on the right, with no acute lung pathology.

On physical examination, bilateral air entry was present without crackles or wheezes. Heart sounds were best heard in the right fifth intercostal space at the midclavicular line, suggestive of dextrocardia. There was no digital clubbing. A chest radiograph (Figure 1) demonstrated dextrocardia without radiographic evidence of active pulmonary infection. Oral amoxicillin-clavulanate was initiated empirically, considering the history of recurrent productive cough and hemoptysis, suggestive of possible bacterial bronchial infection. Oral tranexamic acid was prescribed for hemoptysis, along with an antitussive syrup (chlorpheniramine and dextromethorphan). A montelukast-fexofenadine combination was given to address possible allergic airway inflammation. The patient was advised to monitor symptoms and return for follow-up if necessary.

**Figure 2:**
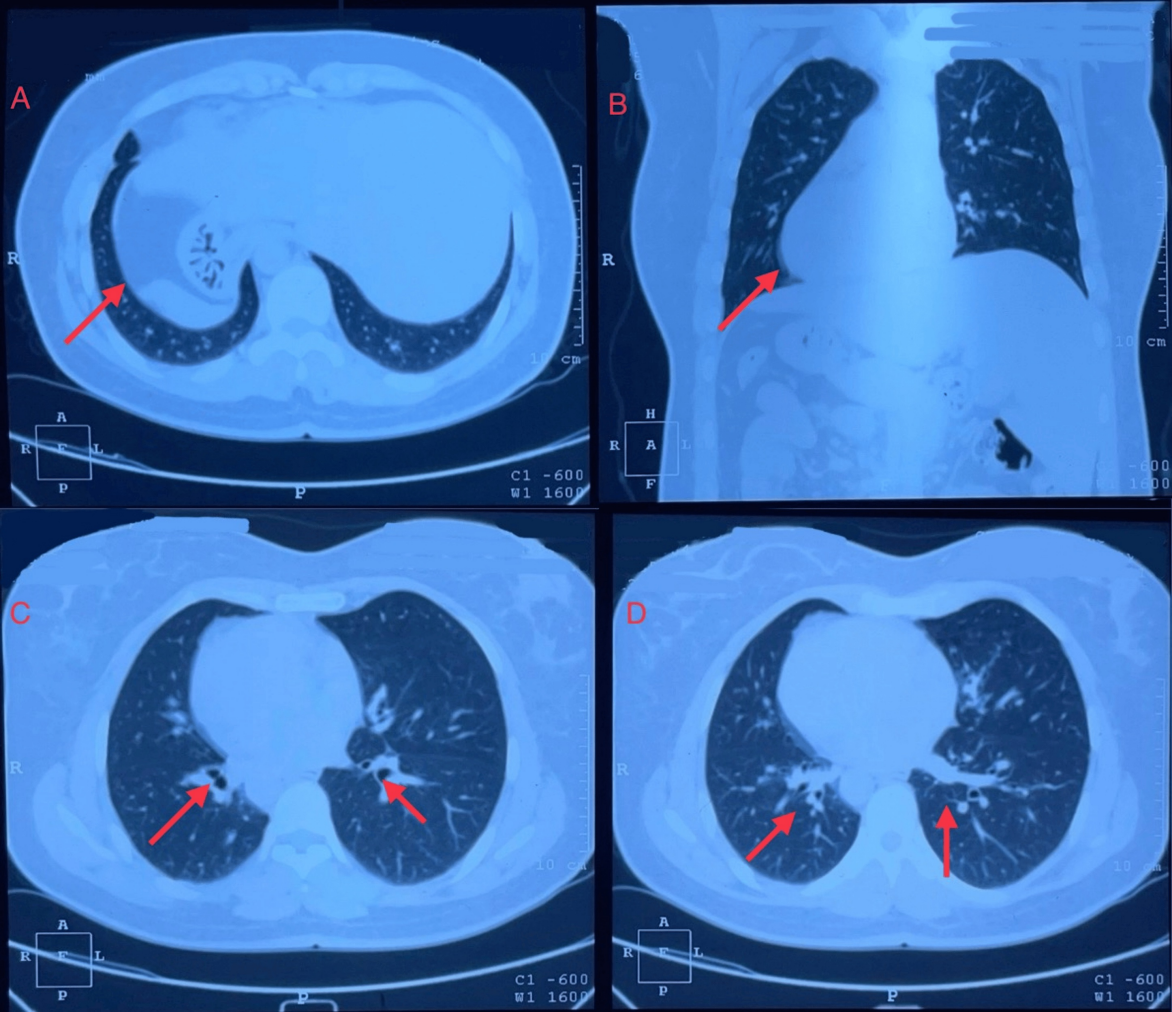
High-resolution computed tomography (HRCT) of the chest. HRCT of the chest shows in panel A (red arrow) segmental areas of relative hypodensity compared to the adjacent lung parenchyma, indicating mosaic attenuation, suggestive of air trapping or regional perfusion abnormalities. The red arrow in panel B indicates the rightward orientation of the cardiac apex, consistent with dextrocardia. Red arrows in panel C point to thickened bronchial walls, characteristic of bronchiectasis. Red arrows in panel D demonstrate the tree-in-bud pattern, indicative of bronchiolar inflammation and small airway disease.

She re-presented to the health center three weeks after the previous visit with an exacerbation of symptoms. On examination, she was afebrile, with stable vital signs. She was referred to a diagnostic center for imaging, where a high-resolution computed tomography (HRCT) scan of the chest (Figure 2) revealed bronchiectasis in the left lower lobe and middle lobe, along with a tree-in-bud pattern in the right lower lobe, suggestive of endobronchial spread of infection. Mirror-image bronchial anatomy suggestive of situs inversus was also noted. Given the presentation of productive cough, intermittent hemoptysis, and a history of recurrent respiratory infections, an acute exacerbation of chronic bronchiectasis with possible secondary bacterial infection was suspected. Oral cefpodoxime-clavulanate was prescribed to cover common respiratory pathogens associated with bronchiectasis exacerbations. An antihistamine-antitussive combination (chlorpheniramine with levodropropizine) was added to help reduce cough frequency, improve patient comfort, and enhance sleep quality.

**Figure 3:**
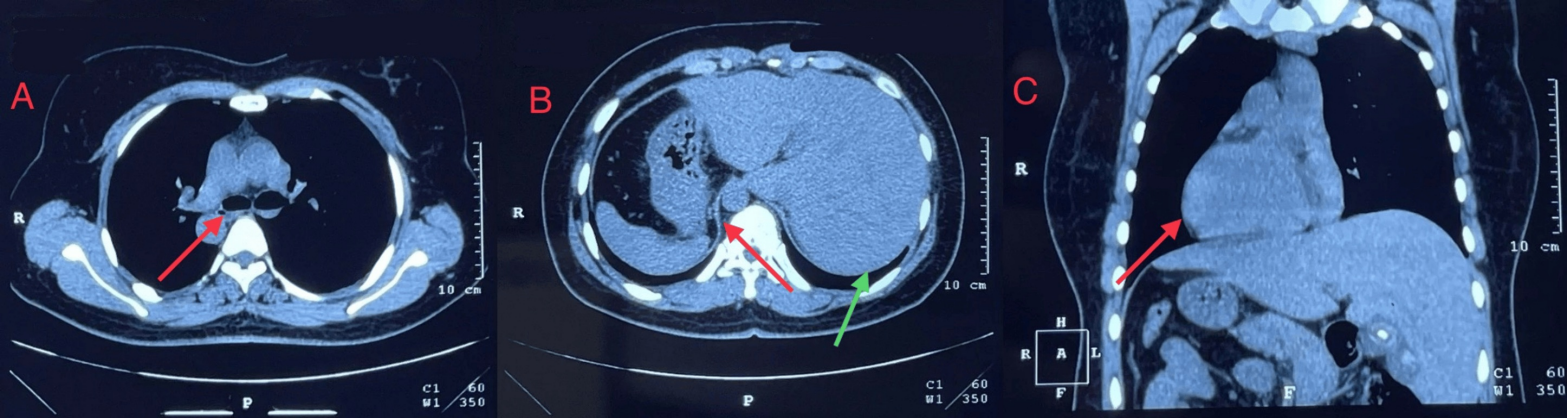
Non-contrast computed tomography (NCCT) scan of the thorax and upper abdomen. NCCT of the thorax and upper abdomen in panel A (red arrow) shows enlarged bronchi with thickened walls, consistent with bronchiectasis. The red arrow in panel B shows a hypodense gas shadow (gastric bubble) appearing on the right, while the green arrow in panel B shows liver parenchyma on the left, confirming situs inversus. Panel C shows the liver on the left and stomach gas shadow on the right, with the red arrow shows cardiac silhouette on the right, further confirming situs inversus totalis.

Concurrently, and in consultation with the treating physician, a non-contrast computed tomography (CT) scan of the chest (Figure 3) was performed at the diagnostic center. It demonstrated subsegmental consolidation, mild fibrotic changes, and traction bronchiectasis in the left middle lobe, raising the possibility of pulmonary tuberculosis versus active infection. Situs inversus totalis was also confirmed, with the heart, liver, and gallbladder located on the left, and the stomach, spleen, and pancreas on the right. The right lung had two lobes and the left lung had three, consistent with a mirror-image arrangement of normal lung anatomy. Sputum samples were sent for acid-fast bacilli (AFB) staining, cartridge-based nucleic acid amplification test (CB-NAAT), and bacterial culture and sensitivity. Both CB-NAAT and AFB staining were negative. Sputum culture yielded only normal upper respiratory tract flora. The negative result of AFB staining implied that mycobacterial infections, including tuberculosis (TB) and non-tuberculous mycobacteria (NTM), are less likely. Combined with the negative result of CB-NAAT, the suspicion of TB was much lower. Culture for mycobacterial infection, including TB and NTM, was not performed due to limited access to appropriate resources.

**Figure 4:**
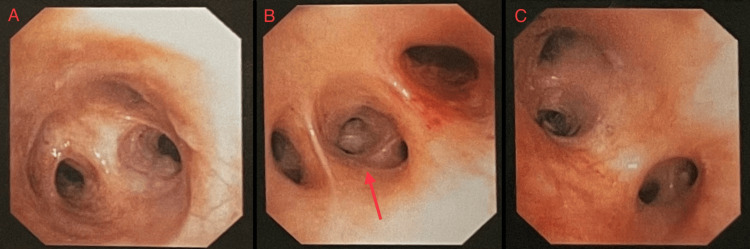
Bronchoscopy. Flexible bronchoscopy of the tracheobronchial tree revealed mirror-image anatomy consistent with situs inversus. Panel A shows the tracheal bifurcation at the carina, with clear visualization of the right and left main bronchi. Panel B demonstrates the left main bronchus, which in situs inversus gives rise to three lobar branches (upper, middle, and lower). The red arrow in panel B highlights the segmental branches arising from the left middle lobe bronchus (B4–B5), along with the superior segment branch (S6) and basal branches (B7–B10), thereby confirming mirror-image anatomy. Panel C shows the right main bronchus dividing into two lobar branches (upper and lower), analogous to the branching pattern of the physiological left lung, further validating reversed bronchial anatomy.

A diagnostic bronchoscopy was planned (Figure 4). The procedure was performed using a flexible bronchoscope. Topical anesthesia was achieved with 2% and 4% Xylocaine, and no sedation was administered. The scope was introduced via the nasal route. The upper respiratory tract appeared normal. The vocal cords were mobile and structurally intact. The trachea was visualized and appeared slightly distorted. Carina was sharp. The tracheobronchial tree revealed a mirror-image anatomy consistent with situs inversus. At the level of the carina, the trachea bifurcated into the right and left main bronchi. The left main bronchus was observed to branch into three divisions, while the right main bronchus divided into two lobar branches (upper and lower), further confirming the reversed bronchial anatomy.

Baseline investigations were ordered, including complete blood count, liver and kidney function tests, random blood sugar, serum electrolytes, and screening for common viral infections. Following bronchoscopy, the bronchoalveolar lavage (BAL) fluid Gram stain showed Gram-positive diplococci. BAL culture demonstrated growth of *Pseudomonas aeruginosa*, while cytology was negative for malignant cells (Table 1).

**Table 1:** Laboratory test results with corresponding reference ranges. SGOT: serum glutamic oxaloacetic transaminase; SGPT: serum glutamic pyruvic transaminase; ELISA: enzyme-linked immunosorbent assay.

Test	Result	Reference range
Hemoglobin (Hb)	11.2 g/dL	12–16 g/dL (female)
Total leukocyte count (TLC)	12,600/mm³	4,000–11,000/mm³
Neutrophil percentage	78%	40–70%
Lymphocyte percentage	17%	20–40%
Eosinophil percentage	1%	1–6%
Platelet count	310,000/mm³	150,000–400,000/mm³
Erythrocyte sedimentation rate (ESR)	45 mm/hr	<20 mm/hr (female)
C-reactive protein (CRP)	28 mg/L	<6 mg/L
Random blood sugar (RBS)	98 mg/dL	70–140 mg/dL
Serum creatinine	0.9 mg/dL	0.6–1.1 mg/dL
Blood urea nitrogen (BUN)	12 mg/dL	7–20 mg/dL
Aspartate transaminase (AST/SGOT)	32 U/L	5–40 U/L
Alanine transaminase (ALT/SGPT)	28 U/L	7–56 U/L
Alkaline phosphatase (ALP)	89 U/L	40–129 U/L
Serum sodium (Na⁺)	139 mmol/L	135–145 mmol/L
Serum potassium (K⁺)	4.1 mmol/L	3.5–5.0 mmol/L
Serum chloride (Cl⁻)	102 mmol/L	98–107 mmol/L
Human immunodeficiency virus I & II ELISA (HIV)	Non-reactive	–
Hepatitis B surface antigen (HBsAg)	Negative	–
Hepatitis C virus antibody (Anti-HCV)	Negative	–
Acid-fast bacilli smear (AFB – sputum)	Negative	–
Cartridge-based nucleic acid amplification test (CB-NAAT)	Negative	–
Sputum culture	Normal flora	–
Bronchoalveolar lavage Gram stain (BAL Gram)	Gram-positive diplococci	–
Bronchoalveolar lavage culture (BAL C/S)	Pseudomonas aeruginosa	–
Bronchoalveolar lavage cytology (BAL cytology)	Negative	–

On account of these, a presumptive diagnosis of Kartagener syndrome was made on the basis of clinical presentation and imaging features, the complete diagnosis being “Left lower lobe bronchiectasis with secondary infection (*Pseudomonas aeruginosa*) with situs inversus; Kartagener syndrome.” The patient was placed on inhaled steroids, antibiotics (intravenous meropenem and moxifloxacin), mucolytics, and bronchodilators. She had a fair response to treatment and still comes for follow-up clinic visits, and she is being monitored for possible complications.

## Discussion

Kartagener syndrome (KS), a subtype of PCD, presents a significant diagnostic challenge, particularly in resource-limited settings. The diagnosis is typically initiated by clinical suspicion based on the characteristic triad of situs inversus, chronic sinusitis, and bronchiectasis, often manifesting since childhood. Additional supportive criteria include the following: (a) situs inversus in the patient or siblings, (b) immotile but viable spermatozoa, (c) impaired mucociliary clearance, and (d) ultrastructural abnormalities of cilia on electron microscopy [4].

Our patient exhibited classical features suggestive of KS, including situs inversus totalis, recurrent lower respiratory tract infections, radiologically confirmed bronchiectasis, and a documented episode of pneumonia. Although she reported no history of infertility, female fertility can be preserved in some individuals with partial ciliary function despite underlying ultrastructural defects [5].

In clinical practice, the diagnosis of PCD (and by extension KS) relies on the combination of clinical features and specialized investigations. Screening tests such as nasal nitric oxide measurement (typically low in PCD) and the saccharin test (assessing nasal mucociliary clearance) are helpful but not definitive [6,7]. Confirmatory diagnosis usually requires high-speed video microscopy to evaluate ciliary beat frequency and pattern, along with transmission electron microscopy (TEM) to demonstrate hallmark ultrastructural defects.

In our setting, access to such advanced diagnostics was unavailable, precluding nasal nitric oxide estimation, ciliary biopsy, electron microscopy, high-speed video analysis, genetic testing for PCD-associated mutations, and transmission electron tomography. This raises an important question: Can a diagnosis of KS be made on clinico-radiological grounds alone? In this case, classical clinical features (chronic productive cough, recurrent respiratory infections, and situs inversus), chest radiography confirming dextrocardia, and HRCT revealing bronchiectasis and characteristic pulmonary changes strongly supported the diagnosis. Microbiological cultures further guided therapy by identifying *Pseudomonas aeruginosa*, a known pathogen in bronchiectatic airways. Taken together, these findings provide compelling circumstantial evidence for KS, even though the absence of definitive structural or functional tests renders the diagnosis presumptive.

The differential diagnosis for recurrent respiratory infections with bronchiectasis is broad, including cystic fibrosis, common variable immunodeficiency (CVID), alpha-1 antitrypsin deficiency, and other structural airway anomalies [8]. In our patient, cystic fibrosis was unlikely, given the absence of pancreatic insufficiency, steatorrhea, or growth retardation; CVID was improbable due to the lack of recurrent otitis media or hypogammaglobulinemia. The coexistence of situs inversus and recurrent sinopulmonary infections made KS the most probable diagnosis. This emphasizes a key point for primary care physicians: detailed history-taking and careful evaluation of basic clinical features can guide early recognition and referral, even without access to advanced diagnostics.

Radiology played a pivotal role. HRCT demonstrated central bronchiectasis with mucus plugging, while chest radiography and abdominal ultrasonography confirmed situs inversus. An ECG was performed with reversed lead placement to account for dextrocardia, preventing potential misinterpretation as myocardial injury or conduction abnormality.

Bronchoscopy not only revealed purulent secretions in the left middle lobe bronchial segment but also allowed targeted sampling for AFB smear, culture, fungal studies, CB-NAAT, and cytology. These findings, coupled with the identification of *Pseudomonas aeruginosa*, underscored the contribution of chronic mucociliary dysfunction to disease progression [9].

Long-term management in KS focuses on preventing disease progression and maintaining quality of life. This includes regular monitoring of pulmonary function, chest X-rays, tracking infection frequency, optimizing airway clearance strategies, and periodic hearing assessments. PCD is an autosomal recessive disorder, most often caused by mutations in genes such as DNAI1 and DNAH5 [10]. Therefore, recognizing the inheritance pattern is crucial for family counseling, as there is a 25% recurrence risk in siblings if both parents are carriers.

For primary care physicians working in resource-limited settings, this case underscores several practical considerations. Early recognition of recurrent sinopulmonary infections and unusual anatomical features, such as dextrocardia, can raise clinical suspicion for KS. Even in the absence of advanced diagnostics, basic investigations, including chest X-ray, ECG with attention to lead placement, and HRCT when available, can provide sufficient evidence for a presumptive diagnosis. Targeted microbiological testing can guide appropriate antibiotic therapy and follow-up, while patient education and longitudinal care remain critical for monitoring infections, optimizing airway clearance, and counseling families regarding genetic risks.

In conclusion, while our diagnosis of KS remains presumptive in the absence of confirmatory ultrastructural or genetic testing, the convergence of classical clinical features, radiological evidence, and bronchoscopic findings justified management under this working diagnosis. This case highlights the critical role of clinical acumen in diagnosing rare genetic disorders in low-resource settings and emphasizes the need for broader access to specialized diagnostics. Importantly, management principles remain largely the same regardless of confirmatory testing.

## Conclusions

Kartagener syndrome remains a diagnostic challenge, especially in resource-limited settings where confirmatory tests may not be readily available. In such scenarios, a strong clinical suspicion, supported by radiological findings, can guide diagnosis and management. This case highlights the importance of maintaining a high index of suspicion for primary ciliary dyskinesia in patients presenting with recurrent respiratory tract infections and dextrocardia. Prompt recognition and early treatment of infections, along with multidisciplinary follow-up, can significantly reduce complications and improve long-term outcomes. Our case underlines the need to rely on clinico-radiological diagnosis when diagnostic infrastructure is lacking, while also advocating for improved access to specialized testing for definitive confirmation.

## References

[1] Dhar DK, Ganguly K, Alam S, Hossain A, Sarker U, Das B, Haque MJ (2009). Kartagener’s syndrome. Mymensingh Med J.

[2] Skeik N, Jabr FI (2011). Kartagener syndrome. Int J Gen Med.

[3] Shapiro AJ, Davis SD, Polineni D (2018). Diagnosis of primary ciliary dyskinesia. An official American Thoracic Society clinical practice guideline. Am J Respir Crit Care Med.

[4] Mishra M, Kumar N, Jaiswal A, Verma AK, Kant S (2012). Kartagener's syndrome: a case series. Lung India.

[5] Halbert SA, Patton DL, Zarutskie PW, Soules MR (1997). Function and structure of cilia in the fallopian tube of an infertile woman with Kartagener's syndrome. Hum Reprod.

[6] Shapiro AJ, Josephson M, Rosenfeld M (2017). Accuracy of nasal nitric oxide measurement as a diagnostic test for primary ciliary dyskinesia. A systematic review and meta-analysis. Ann Am Thorac Soc.

[7] Toro MD, Ortiz E, Marson FA, Pinheiro LM, Toro AA, Ribeiro JD, Sakano E (2023). Cross-sectional evaluation of the saccharin transit time test for primary ciliary dyskinesia: Did we discard this tool too soon?. Sao Paulo Med J.

[8] Panigrahi MK (2014). Common variable immunodeficiency disorder - an uncommon cause for bronchiectasis. Lung India.

[9] Wijers CD, Chmiel JF, Gaston BM (2017). Bacterial infections in patients with primary ciliary dyskinesia: comparison with cystic fibrosis. Chron Respir Dis.

[10] Zariwala MA, Leigh MW, Ceppa F (2006). Mutations of DNAI1 in primary ciliary dyskinesia: evidence of founder effect in a common mutation. Am J Respir Crit Care Med.

